# Adrenal Insufficiency in Patients with Corticosteroid-Refractory Cerebral Radiation Necrosis Treated with Bevacizumab

**DOI:** 10.3390/jcm8101608

**Published:** 2019-10-03

**Authors:** Martin Voss, AbdulAziz Batarfi, Eike Steidl, Marlies Wagner, Marie-Thérèse Forster, Joachim P. Steinbach, Claus M. Rödel, Jörg Bojunga, Michael W. Ronellenfitsch

**Affiliations:** 1 Dr. Senckenberg Institute of Neurooncology, University Hospital Frankfurt, Goethe University, 60590 Frankfurt am Main, Germany; Joachim.Steinbach@med.uni-frankfurt.de (J.P.S.); M.Ronellenfitsch@gmx.net (M.W.R.); 2 University Cancer Center (UCT) Frankfurt, University Hospital Frankfurt, Goethe University, 60590 Frankfurt am Main, Germany; 3 German Cancer Consortium (DKTK), 60590 Frankfurt am Main, Germany; 4 Frankfurt Cancer Institute (FCI), University Hospital Frankfurt, Goethe University, 60590 Frankfurt am Main, Germany; 5 Department of Neurology, University Hospital Frankfurt, Goethe University, 60590 Frankfurt am Main, Germany; AbdulAziz.Batarfi@kgu.de; 6 Institute of Neuroradiology, University Hospital Frankfurt, Goethe University, 60590 Frankfurt am Main, Germany; Eike.Steidl@kgu.de (E.S.); Marlies.Wagner@kgu.de (M.W.); 7 Department of Neurosurgery, University Hospital Frankfurt, Goethe University, 60590 Frankfurt am Main, Germany; Marie-Therese.Forster@kgu.de; 8 Department of Radiotherapy and Oncology, University Hospital Frankfurt, Goethe University, 60590 Frankfurt am Main, Germany; ClausMichael.Roedel@kgu.de; 9 Department of Internal Medicine 1, University Hospital Frankfurt, Goethe University, 60590 Frankfurt am Main, Germany; Joerg.Bojunga@kgu.de

**Keywords:** adrenal insufficiency, Addison’s disease, bevacizumab, cerebral radiation necrosis

## Abstract

Cerebral radiation necrosis is a common complication of the radiotherapy of brain tumours that can cause significant mortality. Corticosteroids are the standard of care, but their efficacy is limited and the consequences of long-term steroid therapy are problematic, including the risk of adrenal insufficiency (AI). Off-label treatment with the vascular endothelial growth factor A antibody bevacizumab is highly effective in steroid-resistant radiation necrosis. Both the preservation of neural tissue integrity and the cessation of steroid therapy are key goals of bevacizumab treatment. However, the withdrawal of steroids may be impossible in patients who develop AI. In order to elucidate the frequency of AI in patients with cerebral radiation necrosis after treatment with corticosteroids and bevacizumab, we performed a retrospective study at our institution’s brain tumour centre. We obtained data on the tumour histology, age, duration and maximum dose of dexamethasone, radiologic response to bevacizumab, serum cortisol, and the need for hydrocortisone substitution for AI. We identified 17 patients with cerebral radiation necrosis who had received treatment with bevacizumab and had at least one available cortisol analysis. Fifteen patients (88%) had a radiologic response to bevacizumab. Five of the 17 patients (29%) fulfilled criteria for AI and required hormone substitution. Age, duration of dexamethasone treatment, and time since radiation were not statistically associated with the development of AI. In summary, despite the highly effective treatment of cerebral radiation necrosis with bevacizumab, steroids could yet not be discontinued due to the development of AI in roughly one-third of patients. Vigilance to spot the clinical and laboratory signs of AI and appropriate testing and management are, therefore, mandated.

## 1. Introduction

Cerebral radiation necrosis is a frequent complication of current treatment algorithms for malignant brain tumours [1]. Radiotherapy is an integral part of first-line therapy for primary brain tumours like malignant gliomas as well as brain metastases [2,3]. Because the majority of malignant brain tumours are incurable, recurrent disease is almost always inevitable and a second course of radiotherapy can be considered under some circumstances [4], which further increases the risk of cerebral radiation necrosis. Pathophysiologically, cerebral radiation necrosis is characterized by capillary collapse and liquefaction necrosis of brain tissue, which causes an inflammation and vascular endothelial growth factor (VEGF) A-mediated disruption of the blood–brain barrier (BBB) [1,5,6]. Local inflammation can additionally cause necrotic areas to spread and the associated brain edema can greatly exceed the area of BBB disruption. Therefore, cerebral radiation necrosis can cause significant morbidity.

Established therapy for cerebral radiation necrosis is the administration of high-dose corticosteroids [7]. The most commonly employed dexamethasone is a high-potency, long-acting corticosteroid with a biological half-life of 36 to 54 h, which causes profound suppression of the hypothalamus–pituitary–adrenal hormone axis [8]. Patients, especially those on long-term treatment, frequently experience several side effects including weight gain, body edema, skin thinning, striae rubrae, proximal myopathy, steroid-induced diabetes, sleep disturbance, mood changes and sometimes steroid psychosis or depression, osteoporosis, thrombosis, and infections [9]. Bevacizumab is an antibody targeting VEGF-A as a mediator of angiogenesis [10] and established targeted therapeutic approach in some cancer entities including breast and colorectal cancer [11,12]. Much hope was therefore placed in its possible efficacy in glioblastoma (GB). While the first phase II trial of bevacizumab and irinotecan in recurrent glioblastoma with dramatic improvement in MRI presentation (at least a partial response in 63% of patients) sparked enthusiasm [10], three subsequent phase III trials of first-line therapy failed to show any prolongation of overall survival [13,14,15]. However, similar MRI improvements with reduced gadolinium contrast enhancement had been observed in these studies [13,14,15], revealing the ability of bevacizumab to reduce the permeability of the BBB without a significant anti-GB effect. As a consequence of the tightening of the BBB, bevacizumab also allowed reducing the corticosteroid doses reported, e.g., in the AVAglio trial (BO21990) [15]. This effect of bevacizumab has been used clinically in small patient collectives as a treatment option for cerebral radiation necrosis [16,17,18]. However, bevacizumab has not been approved by the European Medicines Agency (EMA) for this indication. Nevertheless, when dexamethasone has to be discontinued as a treatment for patients with cerebral radiation necrosis due to adverse or insufficient antiedematous effects, bevacizumab is an option as part of an individual, off-label therapeutic approach that frequently allows the tapering off of parallel dexamethasone. Since such patients have commonly been treated with dexamethasone for weeks or months, consecutive adrenal insufficiency (AI) has to be considered. The clinical symptoms of AI are nonspecific, and symptoms like lethargy, weakness, and nausea can be misinterpreted as consequences of the tumour treatment or the tumour itself. It may also be challenging to differentiate between AI occurring as a consequence of terminated dexamethasone treatment, which should be substituted with hydrocortisone, and the recurrence of cerebral edema, which is best treated with dexamethasone. In order to evaluate the frequency of AI in brain tumour patients treated with dexamethasone, we chose a collective of bevacizumab-treated patients because corticosteroids can often be terminated and thus basal cortisol can be analysed accurately in this patient collective.

## 2. Experimental Section

We performed a retrospective analysis of patients treated in our clinic between 2016 and 2019 to identify patients with cerebral radiation necrosis who received bevacizumab and who had at least one documented cortisol value. AI was defined by our laboratory when morning (8–10 a.m.) serum cortisol levels were below 7.25 μg/dL (200 nmol/L) [19]. Cerebral radiation necrosis was diagnosed based on the localization of a lesion within a previously irradiated region, compatible MRI findings including no significant increase in perfusion, non-solid morphology, if available no significantly increased metabolism in O-(2-(18F)fluoroethyl)-L-tyrosine (18F-FET) positron emission tomography (PET) as well as follow-up scans compatible with the diagnosis of radiation necrosis. The patient collective was evaluated with regard to histology, patient age at tumour diagnosis, patient age at cortisol analysis, duration and maximum dose of dexamethasone, the need for hydrocortisone substitution, as well as the radiologic response to bevacizumab treatment. MRI scans including axial fluid-attenuated inversion recovery (FLAIR), T2 weighted, and T1 weighted images before and after application of gadolinium-based contrast agent were analysed by an experienced, board-certified neuroradiologist (M.W.). The extent of edema was estimated on the axial FLAIR or T2 weighted sequence. Response to bevacizumab treatment was defined as a reduction of the edema by at least 25% [18]. Additionally, intracranial contrast-enhancing lesions were measured on postcontrast images as a further marker of the disruption of the blood–brain barrier. Partial response was defined as a reduction of contrast enhancement by at least 50%, and complete response by complete absence of contrast enhancement. Progressive disease was defined as an increase of at least 25%.

Statistical analysis: SPSS Statistics Version 22 was used for statistical analysis (IBM, Armonk, NY, United States). Ethics approval was obtained from the ethics committee of the University Hospital Frankfurt; Goethe University (SNO_01-08). This study was performed in accordance with the declaration of Helsinki.

## 3. Results

### 3.1. Successful Treatment of a Patient with Cerebral Radiation Necrosis with Subsequent Adrenal Insufficiency

**Figure 1:**
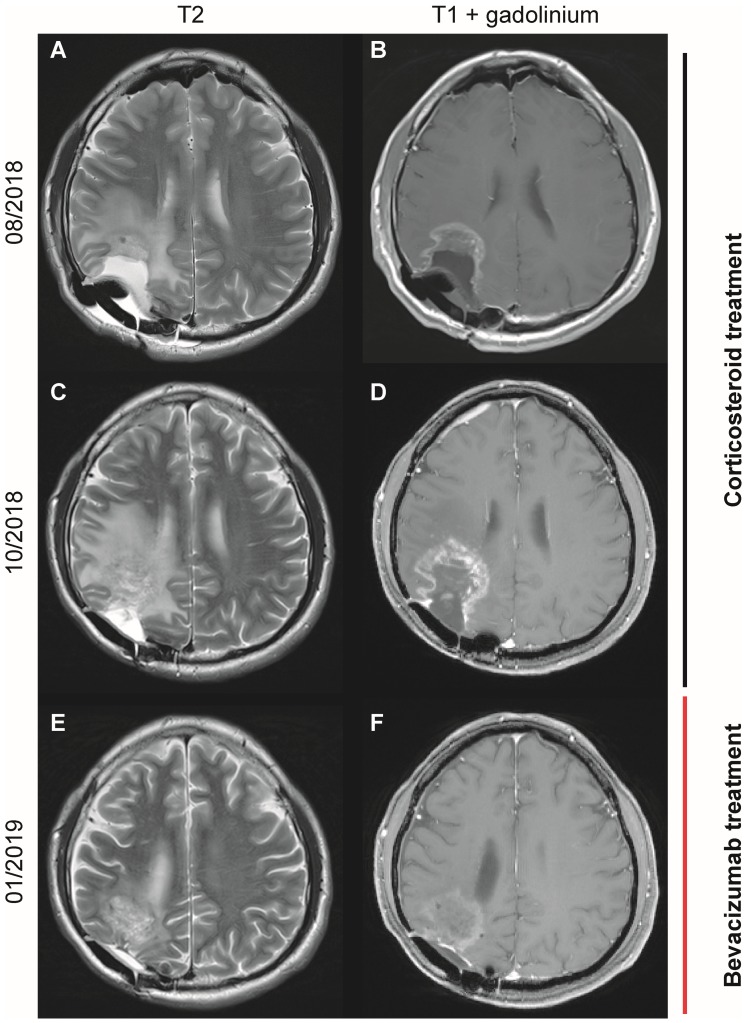
Brain edema and disruption of the blood–brain barrier in an index patient with cerebral radiation necrosis. MRI scans of a 46-year-old patient with glioblastoma of the right parietal lobe (**A**,**C**,**E**): T2 weighted images (WI); (**B**,**D**,**F**): T1 (WI) after intravenous gadolinium). Re-radiation was applied 10 months before the first MRI. (**A**,**B**) MRI shows a non-solid, necrotizing lesion with rim enhancement adjacent to the resection defect with surrounding edema. Due to worsening headache and increased rate of epileptic seizures, dexamethasone was started one month later. (**C**,**D**) the clinical symptoms had declined, yet the MRI shows an increasing extent of the rim-enhancing lesion and of the edema, so therapy with bevacizumab was started one month later. (**E**,**F**) the first follow-up MRI after two infusions of bevacizumab showed a significant reduction of contrast enhancement and edema. Treatment with dexamethasone could be stopped shortly after, but serum analysis revealed an adrenal insufficiency.

The therapeutic potential to treat radiation necrosis is exemplified by one patient who had received a second course of radiation for a recurrent glioblastoma. The initial diagnosis was established by tumour resection in 2016. First-line therapy consisted of radiation therapy with a cumulative dose of 60 Gy with concomitant and adjuvant temozolomide according to the EORTC26981 trial protocol [20]. Five months after the end of chemotherapy, a recurrent tumour was diagnosed and resected. Afterwards, the tumour cavity was treated by another course of radiotherapy with a cumulative dose of 20 Gy. The patient subsequently experienced a worsening of headaches and epileptic seizures. An MRI scan showed an increase of contrast enhancing lesions with corresponding edema and led to treatment of the putative radio necrosis with 8 mg dexamethasone daily. In the course of further treatment, parts of the contrast-enhancing lesions were resected in order to differentiate between tumour recurrence and radiation necrosis, and dexamethasone treatment could be ceased. Cortisol had not been analysed at this time point. Histology showed mainly necrosis and scar tissue. A follow-up MRI again showed progressive edema and contrast enhancement, so that dexamethasone treatment was started once again at a dose of 8 mg per day (Figure 1A,B). Despite this treatment, a follow-up MRI again showed progressive contrast enhancement (Figure 1C,D). As a potential side effect of the dexamethasone therapy, the patient became increasingly aggressive towards family members. A 18F-FET PET scan was compatible with radiation necrosis and therapy with bevacizumab was started. After two infusions of bevacizumab, MRI already confirmed a significant reduction of both contrast enhancement and cerebral edema (Figure 1E,F). Treatment with dexamethasone could be stopped shortly thereafter, but serum analysis revealed severe AI with 0.7 µg/dL cortisol. Thus, substitution with hydrocortisone was started. After the therapy with bevacizumab and the end of dexamethasone, the belligerence and rate of epileptic seizures had significantly improved.

### 3.2. Identification of a Patient Cohort with Cerebral Radiation NECROSIS and Treatment with Bevacizumab

Forty patients with bevacizumab treatment and cerebral radiation necrosis were identified. Routine cortisol testing is not included in institutional guidelines and at least one cortisol analysis was available in 17 of these patients. Fifteen patients (88%) suffered from primary brain tumours (six GBs, five anaplastic astrocytomas, two diffuse astrocytomas, one ependymoma, one anaplastic meningioma). One patient had a radiation necrosis of the frontal lobe after radiation therapy of a paranasal extracranial tumour, and one patient had a tumour of the cervical myelon without histological confirmation of the tumour entity. Patients’ mean age at the time of tumour diagnosis was 43 years (range 20–65), whereas at the time of cortisol analysis patients were 48 years (range 29–67). Six patients were treated with radiation therapy as first-line treatment; 11 patients had repeated radiation treatment of a recurrent tumour prior to bevacizumab. All but two patients were treated with conventional radiation therapy. Radiation doses varied from 54 to 60 Gy for first-line therapy and 20 to 36 Gy for recurrent tumours. One patient treated for meningioma had undergone C12 ion irradiation; another patient suffering from ependymoma had been treated with stereotactic radiosurgery. In three patients, bevacizumab was part of a tumour therapy regimen; the other 14 patients received 1–7 infusions of bevacizumab, specifically as a treatment of radiation necrosis (Table S1).

Bevacizumab was highly effective in reducing local disruption of the BBB and reducing brain edema, as all patients showed a partial response—defined as a reduction of contrast enhancement by at least 50% in the first follow-up MRI. Fifteen of the 17 patients had a reduction of the edema by at least 25%. However, one GB patient 58 years of age receiving treatment for radiation necrosis after re-irradiation with a cumulative dose of 20 Gy suffered a major stroke after seven infusions as a potential bevacizumab side effect. Two patients developed hypertension, which required antihypertensive medication; otherwise, bevacizumab was well tolerated.

### 3.3. Frequent Adrenal Insufficiency in Patients with Corticosteroid-Refractory Cerebral Radionecrosis

**Figure 2:**
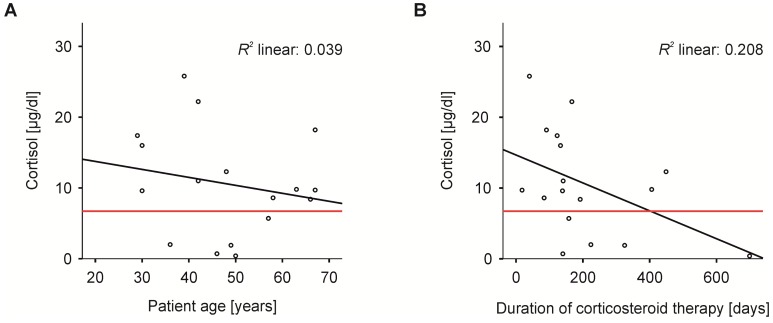
Correlation of cortisol levels with clinical data. Regression analysis of (**A**) patients’ serum cortisol level and their age and (**B**) patients’ serum cortisol level and duration of dexamethasone treatment did not reveal a significant correlation. The red line indicates the lower limit of the normal value of 7.25 μg/dL (200 nmol/L) cortisol. The linear regression line is shown in black.

Treatment with dexamethasone was started at a median of four months (range 0–64) after radiation therapy and continued for a median of 141 days (range 18–699). The median maximum dose of dexamethasone was 8 mg (range 4–100), which was reduced stepwise. Dexamethasone was stopped at a median of 61 days after the start of bevacizumab treatment (range −4–372). In 10 patients, the tapering of dexamethasone consisted of a dose reduction to 0.5–1 mg and subsequent switching to hydrocortisone before the analysis of cortisol. In the seven other patients, dexamethasone doses were reduced to either 0.25 mg daily or 0.5 mg every other day, without switching to hydrocortisone. Cortisol analysis revealed an adrenal suppression (<7.25 μg/dL) in five patients (Figure S1). One of these patients presented only a slight reduction of serum cortisol (patient number 5: cortisol 5.7 μg/dL); the other four patients had a marked pathological cortisol value. Additional confirmation of adrenal function via ACTH stimulation was available in 11 patients (two patients with AI and nine patients without AI, as indicated by basal cortisol values) [21]. Notably, the results of all ACTH stimulation tests matched the results of the basal cortisol analysis. Analysis via *t*-test did not show a significant difference in age, duration of dexamethasone treatment, or time from radiation therapy to start of dexamethasone or to start of bevacizumab between the group of patients with normal adrenal function and the group of patients with AI. The Levene test of variation showed an imbalance of variation for age at cortisol analysis and time from radiation therapy to initiation of dexamethasone/bevacizumab, which could have confounded the results. A comparison of the two groups is given in Table 1. Regression analysis did not show a correlation between cortisol value and duration of dexamethasone treatment or patient age (Figure 2).

## 4. Discussion

We here report that AI is a frequent condition in patients undergoing bevacizumab treatment for cerebral radiation necrosis, which was detectable in approximately 30% of patients after the cessation of dexamethasone. Our study highlights the need for cortisol testing in brain tumour patients, independently of the treatment duration of corticosteroids.

The frequency of AI in our cohort is in line with a study of patients receiving dexamethasone as a supportive drug for high emetogenic chemotherapy, which revealed a rate of AI of 15% [22]. We further confirm the potent effect of bevacizumab in treating radiation necrosis in our cohort of 17 patients, which has previously been published in a randomized cohort of 14 patients [18]. While severe side effects of bevacizumab have been reported when therapy was administered over longer periods and as part of tumour-targeted treatment regimens with chemotherapy, in short-course and reduced-dose treatment, the side effects profile is most likely significantly less severe [13,18,23].

Almost all patients with intracranial tumours receive dexamethasone during their course of treatment. Dexamethasone is used to treat edema during surgery and radiation therapy and as add-on therapy to chemotherapy to improve the neurological deficit or increase drug compatibility. In patients with recurrent tumours, especially after a second round of radiation therapy, dexamethasone is often required at least temporarily. However, there is increasing evidence for a negative influence of corticosteroids on glioma patients [24]. Dexamethasone-induced leukocytosis is associated with shorter survival and increased risk for lymphopenia [25,26,27]. Therefore, the lowest possible dose of dexamethasone should always be administered. Nevertheless, balancing dexamethasone doses with edema-associated morbidity can be challenging in daily practice. Patients treated with bevacizumab as part of a chemotherapy regimen or to treat radiation necrosis are the exception in which dexamethasone can frequently be reduced and eventually stopped. Our retrospective analysis revealed a considerable proportion with AI after prior dexamethasone treatment. This is of the utmost clinical importance because AI symptoms can easily be overlooked. Fatigue is one of the most frequent complaints of tumour patients undergoing radiation therapy, but it can also be indicative of AI. We therefore propose testing for AI when terminating dexamethasone treatment with a low threshold, especially in elderly patients and patients who have received dexamethasone over a long period. Additionally, in times of stress (surgery, infection) or unspecified clinical deterioration, cortisol testing should be included in the clinical workup. Notably, none of the patients in our cohort permanently discontinued corticosteroids without prior evidence of sufficient adrenal function. Therefore, specific clinical symptoms of AI were not detected.

The main limitation of our analysis is the small and select sample size, with potential bias for overrating the frequency of AI with regard to the general population of brain tumour patients. Cortisol analysis is not routinely included in the laboratory workup of glioma patients and was only available in 17 of the 40 initially identified patients with bevacizumab treatment for cerebral radiation necrosis (Figure S1). The small sample size may also be the cause of the rather unexpected lack of a significant correlation between the duration of dexamethasone treatment and AI. However, this result is in line with findings in other studies. AI was common in patients with corticosteroid treatment for glomerular disease, but was not predicted by daily dose or duration of treatment [28]. In patients with rheumatoid arthritis treated with corticosteroids, the duration of treatment was also not significantly associated with AI [29].

Another limitation is the bias of a retrospective analysis. It is possible that cortisol was only determined when a higher risk of AI was anticipated and, thus, the proportion of AI may be overestimated. However, cortisol testing is not a common element in routine serum analyses in nonpituitary brain tumour patients, and therefore AI may be greatly underdiagnosed. Further prospective data collection is necessary to estimate the true rate of AI in glioma patients.

## Figures and Tables

**Table 1:** Comparison of the patients with and without adrenal insufficiency.

	Normal Adrenal Function	Adrenal Insufficiency	*p*-Values
Number of patients	12	5	
Age at tumour diagnosis (years)	44 ± 15	40 ± 11	*p* = 0.587
Age at cortisol analysis (years)	48 ± 15	48 ± 8	*p* = 0.885
Time from end of radiation therapy to start of dexamethasone (months)	6 ± 8	17 ± 27	*p* = 0.397
Time from end of radiation therapy to start of bevacizumab (months)	9 ± 8	22 ± 28	*p* = 0.326
Duration of dexamethasone treatment (days)	165 ± 132	309 ± 230	*p* = 0.120

Data are displayed as mean ± standard deviation. *p*-values were calculated using an independent samples *t*-test.

## Supplementary Materials

The following are available online at https://www.mdpi.com/2077-0383/8/10/1608/s1, Supplementary Table S1. Patient collective. Supplementary Figure S1: Consort diagram of the study population.
