# Supplementary material for: Adrenal Insufficiency in Patients with Corticosteroid-Refractory Cerebral Radiation Necrosis Treated with Bevacizumab
Source: J Clin Med. 2019 Oct 3;8(10):1608. doi: 10.3390/jcm8101608 (PMC6832264; doi:10.3390/jcm8101608)
Supplement: Supplementary file 1 [file jcm-08-01608-s001.pdf]

Supplementary Table 1. Patient collective.

Patient	Diagnosis	Age at cortisol analysis	Radiation therapy before bevacizumab	Dose of radiation therapy [Gy]	Re-irradation?	Duration of dexamethasone [days]	Maximum dose of dexamethasone [mg]	Time from end of radiation to start of bevacizumab [months]	Number of bevacizumab infusions before cortisol analysis	Cortisol [µg/dl]	Response in T1 + gadolinium	Response of edema
1	Anaplastic Meningioma	67	C12 ion radiotherapy	68 (incl. Boost)	no	91	24	8	4	18.2	PR	no
2	Glioblastoma	46	Conventional radiotherapy	20.0	yes	140	40	11	4	0.7	PR	yes
3	Glioblastoma	58	Conventional radiotherapy	20.0	yes	84	6	1	7	8.6	PR	yes
4	Ependymoma	67	Stereotactic radiosurgery	14.0	yes	18	8	3	4	9.7	PR	yes
5	Anaplastic Astrocytoma	57	Conventional radiotherapy	20.0	yes	158	16	2	10	5.7	PR	yes
6	Anaplastic Astrocytoma	30	Conventional radiotherapy	27.5	yes	139	4	9	4	9.6	PR	yes
7	Anaplastic Astrocytoma	29	Conventional radiotherapy	60.0	no	123	12	25	5	17.4	PR	yes
8	Diffuse Astrocytoma	50	Conventional radiotherapy	39.0	yes	699	8	28	>20	0.4	PR	yes
9	Diffuse Astrocytoma	63	Conventional radiotherapy	20.0	yes	406	4	8	5	9.8	PR	no
10	Anaplastic Astrocytoma	39	Conventional radiotherapy	35.0	yes	40	12	5	1	25.8	PR	yes
11	Glioblastoma	42	Conventional radiotherapy	35.0	yes	167	100	8	1	22.2	PR	yes
12	Small cell neuroendocrine carcinoma	36	Conventional radiotherapy	30.0	yes	224	8	68	4	2.0	PR	yes
13	Anaplastic Astrocytoma	30	Conventional radiotherapy	35.0	yes	133	16	3	4	16.0	PR	yes
14	Glioblastoma	48	Conventional radiotherapy	60.0	no	449	8	2	27	12.3	PR	yes
15	Tumor of the cervical myelon	49	Conventional radiotherapy	54.0	no	325	8	3	4	1.9	PR	yes

16	Glioblastoma	42	Conventional radiotherapy	60.0	no	141	12	5	4	11.0	PR	yes
17	Glioblastoma	66	Conventional radiotherapy	60.0	no	192	6	25	3	8.4	PR	yes

Patient 8 had been treated with bevacizumab in another clinic and number of infusions prior to cortisol analysis can only be estimated. Criteria for response of are described in material and methods. Partial response is abbreviated as PR.

Supplementary Figure 1.

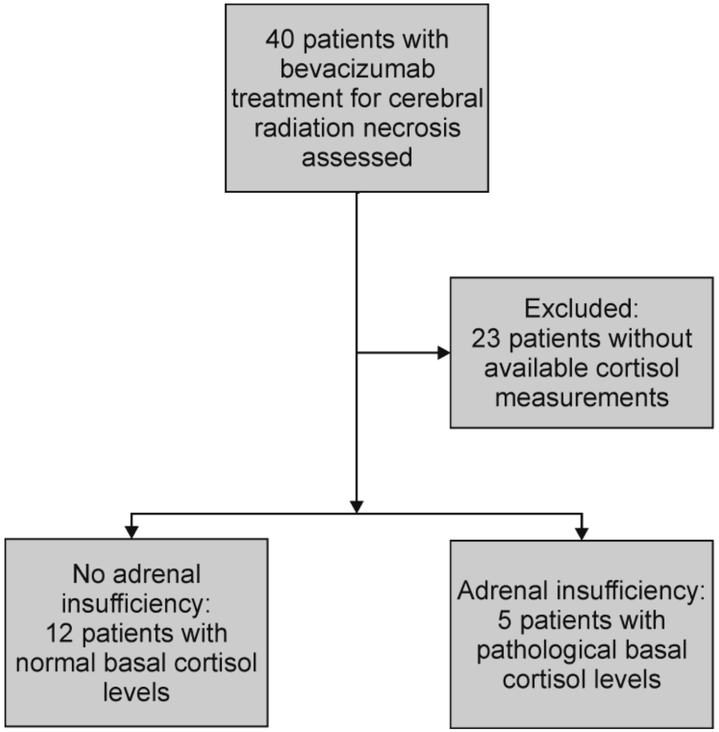

Supplementary Figure 1. Consort diagram of the study population. 40 patients with bevacizumab treatment for cerebral radiation necrosis were identified. Cortisol analyses were available in 17 patients and showed pathological concentrations indicating adrenal insufficiency in 5 patients.
